# Genomic analysis of *Klebsiella pneumoniae* isolates from Malawi reveals acquisition of multiple ESBL determinants across diverse lineages

**DOI:** 10.1093/jac/dkz032

**Published:** 2019-02-18

**Authors:** Patrick Musicha, Chisomo L Msefula, Alison E Mather, Chrispin Chaguza, Amy K Cain, Chikondi Peno, Teemu Kallonen, Margaret Khonga, Brigitte Denis, Katherine J Gray, Robert S Heyderman, Nicholas R Thomson, Dean B Everett, Nicholas A Feasey

**Affiliations:** 1 Malawi-Liverpool-Wellcome Trust Clinical Research Programme, Blantyre, Malawi; 2 Centre for Tropical Medicine and Global Health, Nuffield Department of Medicine, University of Oxford, Oxford, UK; 3 Mahidol-Oxford Tropical Medicine Research Unit, Bangkok, Thailand; 4 College of Medicine, University of Malawi, Blantyre, Malawi; 5 Quadram Institute Bioscience, Norwich, UK; 6 Wellcome Sanger Institute, Hinxton, Cambridge, UK; 7 Liverpool School of Tropical Medicine, Liverpool, UK; 8 Division of Infection and Immunity, University College London, London, UK; 9 London School of Tropical Medicine, London, UK; 10 University of Edinburgh, Edinburgh, UK

## Abstract

**Objectives:**

ESBL-producing *Klebsiella pneumoniae* (KPN) pose a major threat to human health globally. We carried out a WGS study to understand the genetic background of ESBL-producing KPN in Malawi and place them in the context of other global isolates.

**Methods:**

We sequenced genomes of 72 invasive and carriage KPN isolates collected from patients admitted to Queen Elizabeth Central Hospital, Blantyre, Malawi. We performed phylogenetic and population structure analyses on these and previously published genomes from Kenya (*n* = 66) and from outside sub-Saharan Africa (*n* = 67). We screened for presence of antimicrobial resistance (AMR) genetic determinants and carried out association analyses by genomic sequence cluster, AMR phenotype and time.

**Results:**

Malawian isolates fit within the global population structure of KPN, clustering into the major lineages of KpI, KpII and KpIII. KpI isolates from Malawi were more related to those from Kenya, with both collections exhibiting more clonality than isolates from the rest of the world. We identified multiple ESBL genes, including *bla*_CTX-M-15_, several *bla*_SHV_, *bla*_TEM-63_ and *bla*_OXA-10_, and other AMR genes, across diverse lineages of the KPN isolates from Malawi. No carbapenem resistance genes were detected; however, we detected IncFII and IncFIB plasmids that were similar to the carbapenem resistance-associated plasmid pNDM-mar.

**Conclusions:**

There are multiple ESBL genes across diverse KPN lineages in Malawi and plasmids in circulation that are capable of carrying carbapenem resistance. Unless appropriate interventions are rapidly put in place, these may lead to a high burden of locally untreatable infection in vulnerable populations.

## Introduction

*Klebsiella pneumoniae* (KPN) is an opportunistic pathogen responsible for a wide range of hospital-associated (HA) infections, mostly in immunocompromised individuals.^1–3^ KPN is also increasingly implicated in community-acquired (CA) infections in healthy individuals.^1^^,^^4^ The disease syndromes associated with KPN include pneumonia, bacteraemia, urinary tract infections, wound or soft tissue infections and liver abscess.^1^ In the USA, KPN was identified as a leading cause of HA infections and was estimated to cause 8.0% of all HA infections, while in the UK, KPN was implicated in 4.7%–6.0% of all bacterial infections.^5^ Sparse data are available from sub-Saharan Africa (sSA), but published studies do suggest KPN is responsible for higher proportions of HA infections in this region than those reported in industrialized countries, especially among children under 5 years old. In South Africa, KPN caused 22.0% of HA bacteraemia among neonates, whereas in Kenya, KPN was estimated to be responsible for 20.0% of HA bacteraemia.^6^^,^^7^ Additionally, KPN is consistently reported as a common cause of CA infection in sSA. We previously reported that KPN caused 4.4% of CA bacteraemia over a period of 20 years in Malawi and is becoming an increasingly important cause of bacteraemia in children aged <5 years and the elderly.^4^^,^^8^

Health agencies such as WHO and CDC have identified KPN as an urgent threat to human health due to its ability to rapidly acquire and stably express resistance to multiple antimicrobial classes, including antimicrobial agents of last resort.^1^^,^^9^^,^^10^ This is particularly challenging in sSA, where the available antimicrobial classes are fewer than in high-income settings, and cephalosporins are often the antimicrobial of choice, so ESBL-producing pathogens present an extreme therapeutic challenge.

Recent WGS studies of global and national collections of KPN have offered a glimpse of the diversity and antimicrobial resistance (AMR) associated with this pathogen.^9^^,^^10^ This includes identification of hypervirulent and MDR clones such as clonal groups CG258 and CG14, which have caused hospital outbreaks in several countries in Europe and Asia.^11–13^ Such studies have further helped us to understand the mechanisms through which AMR spreads, whereby both horizontal gene transfer (HGT) and clonal expansions have been identified as the main mechanisms of AMR spread across various KPN lineages.^9–11^^,^^14^^,^^15^ Despite this increasing knowledge of the diversity of KPN globally, few studies have included isolates from sSA and there is, therefore, limited understanding of the genomic background of AMR in KPN in the region. In Malawi, the ESBL-producing proportion of KPN has increased to over 90.0% concurrent with ceftriaxone becoming the antimicrobial agent of choice for treating severe bacterial infections.^4^ Such very high rates could suggest either rapid expansion of a single ESBL-producing KPN clone or high selection pressure resulting from the increased use of third-generation cephalosporins driving the spread of ESBL genes across almost all available KPN lineages. We carried out a WGS study using KPN isolates from a single site in Malawi to understand the genetic background of ESBL-producing strains in this setting and place them in the context of the global population structure of KPN.

## Methods

### Study setting and isolates

We used samples collected as part of routine bacteraemia and meningitis surveillance at Queen Elizabeth Central Hospital (QECH), Blantyre, Malawi and archived at the Malawi-Liverpool-Wellcome Trust Clinical Research Programme (MLW). Isolates were selected with the aim of maximizing AMR diversity and included invasive isolates (*n* = 59), from blood and CSF, and carriage isolates from rectal swabs (*n* = 13). Blood and CSF samples were taken from adult and paediatric patients presenting to QECH between 1996 and 2014, within 48 h of admission to hospital, and hence isolates were considered to be CA. Rectal swabs for carriage isolates were collected from adult patients with no suspected bacterial infection during a prevalence survey over a period of two weeks in 2009. Antimicrobial susceptibility tests were performed by the disc diffusion method following BSAC guidelines (www.bsac.org.uk). Isolates were routinely tested for susceptibility to representatives of six commonly used antimicrobial classes, namely ampicillin, cotrimoxazole, chloramphenicol, gentamicin, ceftriaxone/cefpodoxime and ciprofloxacin. Isolate-specific year and clinical site of isolation, phenotypic AMR profiles and patient age categories are presented in Table S1 (available as Supplementary data at *JAC* Online). Whole genome DNA extraction for selected isolates was done at MLW laboratories using the QIAGEN Universal Biorobot (Hilden, Germany) following the manufacturer’s instructions.

**Table 1.** Mean number of pairwise single nucleotide variants by KPN lineage and origin of isolates

	Mean pairwise SNP difference (×10^3^)		
Origin	KpI	KpII	KpIII
Malawi	11.6	14.7	14.7
Kenya	11.6	73.1	12.7
Outside sSA	12.8	29.8	14.1

### WGS, de novo assembly and sequence annotation

Genomic DNA was sequenced at the Wellcome Sanger Institute using the Illumina HiSeq 2000 platform (Illumina, Inc., San Diego, CA, USA) to generate paired-end sequence reads of 100 bp length. Velvet v1.2.09^16^ was used for *de novo* assembly of sequence reads into contiguous sequences following the pipeline by Page *et al.*^17^ Sequence assemblies were annotated *in silico* using the Prokka v1.11 bacterial annotation pipeline.^18^ Raw sequence data were deposited in the European Nucleotide Archive (ENA) and ENA accession numbers are included in Table S1.

### Published genome datasets

In order to place the genetic diversity and population structure of the Malawian KPN isolates in a global context, we analysed our sequenced genomes together with other previously sequenced KPN genomes from around the world. We selected genome sequences from a study that defined the global population structure of KPN and another that investigated genomic epidemiology of KPN in Kenya.^7^^,^^9^ The global KPN study identified that KPN belongs to three major lineages, namely KpI, KpII and KpIII, and we used cluster random sampling to select 67 human invasive and carriage isolates from each of those phylogroups in this global collection. From the Kenyan collection, 66 isolates were selected systematically as isolate identifiers were not matched to phylogroups. A list of ENA accession numbers for the selected global and Kenyan isolates are included in Table S2.

**Table 2.** Recombination statistics of KPN ST14 and ST15 (CC14) isolates from Malawi. Isolates were mapped to the chromosome sequence of KPN MLST15 reference strain (Genbank Accession number CP022127)

Isolate ID	ST	No. of recombination sites	No. of recombination blocks	Recombination sites/mutation (*r/m*)	Bases mapped
D25597	ST14	0	0	0	5055359
1022430	ST14	0	0	0	5035481
A28	ST14	0	0	0	5002581
8193	ST14	28	1	1.6	5078006
D39172	ST14	347	4	2.4	5021455
1023547	ST14	0	0	0	5035136
D3538	ST14	20	1	0.5	5078081
D44912	ST14	0	0	0	5002012
4604	ST14	0	0	0	5055065
D29665	ST14	0	0	0	5055654
D53369	ST14	1165	9	5.7	5013254
C24a	ST15	823	6	16.1	5164584
1007011	ST15	639	2	11.6	5091879
D25466	ST15	335	4	12.9	5097947

### Phylogeny reconstruction and inference of population structure

We used the Roary pan-genome pipeline^19^ to construct a core genome of the annotated genome assemblies of the 205 isolates included in our analysis. In trading off between identifying a core genome that is representative of all the KPN lineages in this collection and accounting for possible assembly errors, we classified a gene as core if it was conserved in at least 99.0% of the genomes. A core genome alignment was then generated through concatenation of the alignments of orthologous core genes. Based on the core genome alignment, we grouped isolates into unique genome sequence clusters (SCs) using the hierBAPS module in the Bayesian Analysis of Population Structure (BAPS) v.6.0 software.^20^ SNP sites were generated from the core-genome alignment and used to construct a maximum likelihood (ML) phylogenetic tree with RAxML v.7.8.6 under the General Time Reversible (GTR) substitution model with a gamma rate of correction heterogeneity.^21^^,^^22^ The reliability of the inferred branches and branch partitions in the phylogenetic tree was assessed using 100 bootstrap replicates. Raw sequence reads of isolates belonging to clonal complex 14 (CC14) from the Malawian collection were mapped to the MLST15 reference strain (Genbank: CP022127) using SMALT (https://www.sanger.ac.uk/science/tools/smalt-0*)* and we performed recombination analysis on the resulting alignment using Gubbins.^23^

### In silico molecular typing of study isolates

We did molecular characterization of the isolates by MLST^24^ and capsule polysaccharide typing (K-typing). MLST was performed by a BLAST search (100% match identity) of sequence assemblies against the PubMLST to identify the different allelic profiles of each isolate based on seven housekeeping genes including *gapA, infB, mdh, pgi, phoE, rpoB* and *tonB*. Isolates were K-typed using the Kaptive locus typing and variant evaluation tool with k-locus searches performed against the Kaptive KPN k-locus reference database.^25^

### Determination of AMR and plasmid typing

We screened for presence of acquired AMR genes by an automated nucleotide BLAST search of our genome assemblies against a curated ResFinder database.^26^ Presence of a gene in an isolate was confirmed if its assembled sequence had at least 95.0% nucleotide matching identity with a gene in the database for a coverage of at least 90.0%. We analysed translated nucleotide sequence alignments of the *gyrA, gyrB, parC* and *parE* genes to identify specific amino acid mutations that were associated with fluoroquinolone resistance (FQR). *In silico* plasmid typing was also performed by a BLAST search of plasmid replicons against the PlasmidFinder database.^27^ As with the search for the AMR genes, we used thresholds of 95.0% and 90.0% for nucleotide identity match and match length, respectively.

### Statistical analyses

We compared mean pairwise SNP differences between lineages and between places of origin of isolates using *t*-tests. *χ*^2^ tests, where appropriate, or Fisher’s exact tests were used to test for AMR gene–phenotype associations and AMR gene–plasmid associations. Linear regression was performed to model the relationship between time and number of AMR genes per genome. All statistical analyses were performed using the R v.3.3.2 statistical package (https://www.r-project.org/).

## Results

### Genetic diversity of Malawian KPN isolates

Pan-genome analysis of the 205 KPN genome sequences (72 Malawian and 133 previously published) predicted a total of 32629 unique protein-coding sequences (CDSs); 2449 (7.5%) CDSs were identified in ≥99.0% of isolates and so formed the core genome. The accessory genome, comprising the remaining 30180 CDSs identified in <99.0% of the isolates, predominantly comprised genes that were uncommon, with 26815 (88.9%) being present in <15.0% of the isolates and 12438 (40.2%) that were isolate specific.

### Phylogeny and population structure

**Figure 1.**
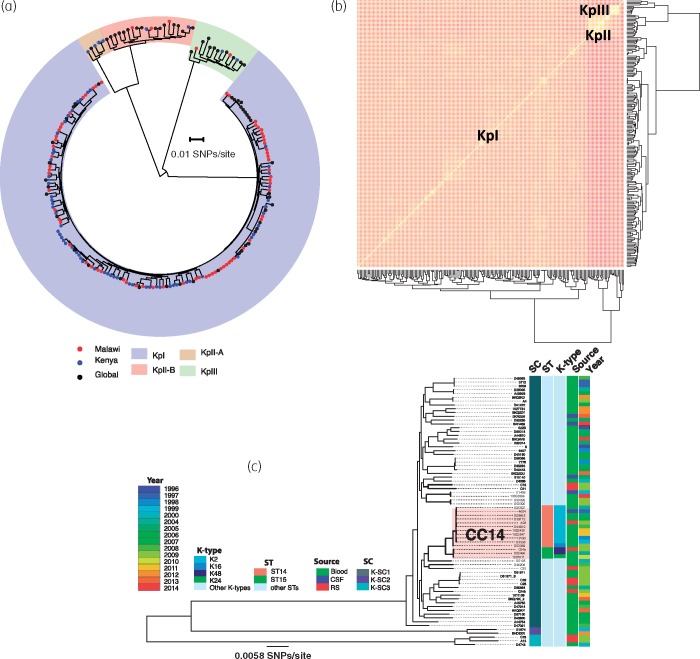
Population structure and genetic diversity of KPN. (a) A phylogenetic tree of Malawian isolates in the context of previously published global KPN isolates constructed from core SNP alignment and rooted at the middle of the branch separating the two most divergent sequences. (b) A heatmap illustrating clustering of Malawian and global KPN isolates by accessory genes. (c) Core-genome phylogenetic tree of KPN isolates from Malawi only, which also shows key STs and K-types and the phylogenetic mixing of isolates from different sources and years of isolation. This figure appears in colour in the online version of *JAC* and in black and white in the print version of *JAC*.

The core genome of all 205 genomes had 307392 SNPs. Phylogenetic and BAPS analyses clustered the isolates into four SCs, which corresponded to the KPN phylogroups KpI (KPN), KpII-A and KpII-B (*Klebsiella quasipneumoniae*) and KpIII (*Klebsiella variicola*) (Figure 1a). The majority of Malawian isolates were KpI [93.1% (67/72)], whereas only three and two isolates were KpII-B and KpIII, respectively. None of the Malawian isolates belonged to the KpII-A cluster. Isolates differed in nucleotide diversity based on the pairwise SNP differences, both by lineage and origin (Table 1). Comparisons of pairwise SNP differences of KpI isolates by origin showed that Malawian and Kenyan isolates had similar nucleotide diversity (Table 1; *P* = 0.7369), which was significantly lower than the global nucleotide diversity (Table 1; *P* < 0.001). There was consistency between clustering of isolates based on shared accessory genes and BAPS SCs based on core SNPs (Figure 1b). This consistency indicates that genetic differences occurring through HGT were not sufficient to disrupt the population structure of KPN either on a local or global scale. Furthermore, it also shows that HGT was more likely to occur between more closely related isolates.

When data on year of isolation and clinical source of the KPN isolates from Malawi were mapped onto a phylogenetic tree of Malawian isolates only, it was apparent that phylogenetic clustering of isolates was independent of either the clinical source or year of isolation (Figure 1c). More importantly, we also noted phylogenetic mixing of carriage isolates with invasive isolates, suggesting a potential role for carriage strains as a reservoir of invasive strains in the Malawian setting.

Over the past two decades, MLST has become one of the most important and commonly used methods for characterizing bacterial strains.^28^ Here we identified STs of the Malawian isolates by *in silico* MLST. Mapping these STs to the phylogenetic tree of Malawian isolates revealed that most SCs were composed of diverse STs. We ran hierBAPS again, but only on the core-genome alignment of KpI SC isolates from Malawi and identified two subclusters; a monophyletic subcluster consisting of mostly ST14 and a few ST15 isolates, and a polyphyletic subcluster containing isolates with high sequence diversity, whose clustering did not reflect a common evolutionary history. ST14 and ST15 belong to KPN CC14 (Figure 1c) and, in this collection, ST14 [11 isolates (15.5%)] was the most common ST whereas ST15 was the third most common ST [3 isolates (4.2%)], behind ST664 [4 isolates (5.6%)]. Except for one isolate, which belonged to the capsular type K16, the rest of the ST14 isolates belonged to the hypervirulent K-type K2 (Figure 1c). Diverse and less frequent serotypes were distributed across the SCs and serotype variations within STs were common (Table S1).

Recombination events contribute substantially to the evolution of many bacteria and may confound phylogenetic reconstruction.^29^ We attempted to elucidate the role of recombination in the evolution of the CC14 isolates, which were the most common and closely related isolates in the Malawian collection. We ran recombination analysis on the genome sequence alignment resulting from mapping the CC14 isolates to the ST15 reference strain (Genbank: CP022127). Despite the reference being more closely related to the ST15 isolates than ST14 isolates, mutations were 15 times more likely to have been acquired through recombination events in ST15 isolates [mean recombination rate (*r/m*) = 13.5] than in ST14 isolates (mean *r/m* = 0.93; Table 2). We ran this analysis again, but even after mapping the CC14 isolates to an independent ST23 reference strain NTUH-K2044 (GenBank accession number AP006725), recombination rates were still higher in ST15 than ST14 (Table S3). The frequency of recombination events in ST14 isolates ranged from zero to nine, whereas in ST15 isolates, the frequency of recombination events per genome ranged between two and six (Table 2). Homologous recombination events have led to the emergence of MDR and hypervirulent clones that have caused hospital outbreaks in other settings, including ST258, which emerged from ST11.^10^ The frequent recombination events in the Malawian ST15 and some ST14 isolates therefore have potential to give rise to epidemics of highly resistant KPN in this setting.

**Table 3.** List of AMR genes identified in genomes of KPN isolates from Malawi

			Prevalence	
AMR gene	Gene description	Resistance	*n*	%
*fosA*	metalloglutathione transferase	fosfomycin	69	95.8
*oqxA*	Oqx efflux pump	fluoroquinolones	67	93.1
*oqxB*	efflux pump	fluoroquinolones	66	91.7
*sul2*	sulphonamide resistance dihydropteroate synthase	sulphonamides/co-trimoxazole	56	77.8
*dfrA*	dihydrofolate reductase	methoxazole/co-trimoxazole	58	80.1
*aac(6′)-lb-cr*	acetyltransferase	aminoglycoside, fluoroquinolones	53	73.6
*bla*_TEM-1_	β-lactamase	aminopenicillins	53	73.6
*catA*	acetyltransferase	chloramphenicol	53	73.6
*strB*	streptomycin phosphotransferase	aminoglycosides	46	63.9
*strA*	streptomycin phosphotransferase	aminoglycosides	46	63.9
*bla*_CTX-M-15_	ESBL	aminopenicillins, cephalosporins	28	38.9
*sul1*	sulphonamide resistance dihydropteroate synthase	sulphonamides/co-trimoxazole	25	34.7
*bla*_SHV-1_	β-lactamase	aminopenicillins	22	30.6
*tet*(D)	tetracycline efflux	tetracyclines	21	29.2
*bla*_SHV-11_	β-lactamase	aminopenicillins	17	23.6
*mphA*	macrolide phosphotransferase	chloramphenicol	16	22.2
*tet*(A)	tetracycline efflux	tetracyclines	12	16.7
*bla*_OXA-1_	β-lactamase	aminopenicillins	11	15.3
*aadA2*	tetracycline efflux	aminoglycosides	11	15.3
*bla*_SHV-28_	β-lactamase	aminopenicillins	10	13.9
*arr*	ADP-ribosylation catalysing enzyme	rifampicin	7	9.7
*alph3*	aminoglycoside phosphotransferase	aminoglycosides	6	8.3
*qnrB*	plasmid-mediated quinolone resistance	fluoroquinolones	6	8.3
*cmlA1*	MFS transporter/chloramphenicol efflux	chloramphenicol	5	6.9
*floR*	transmembrane segments efflux	chloramphenicol/florfenicol	5	6.9
*bla*_OXA-10_	ESBL	aminopenicillins, cephalosporins	5	6.9
*tet*(B)	tetracycline efflux	tetracycline	5	6.9
*qnrs*	plasmid-mediated quinolone resistance	fluoroquinolones	4	5.6
*bla*_SCO-1_	β-lactamase	aminopenicillins	4	5.6
*bla*_OXA-9_	ESBL	aminopenicillins, cephalosporins	4	5.6
*bla*_SHV-12_	ESBL	aminopenicillins, cephalosporins	3	4.2
*bla*_SHV-26_	β-lactamase	aminopenicillins	3	4.2
*bla*_OKPB_	β-lactamase	aminopenicillins	2	2.8
*ereA*	erythromycin esterase	erythromycin	2	2.8
*bla*_SHV-7_	ESBL	aminopenicillins, cephalosporins	2	2.8
*bla*_TEM-63_	ESBL	aminopenicillins, cephalosporins	1	1.4
*bla*_LEN-16_	β-lactamase	aminopenicillins	1	1.4
*bla*_LEN-25_	β-lactamase	aminopenicillins	1	1.4
*bla*_SHV-133_	β-lactamase	aminopenicillins	1	1.4
*bla*_SHV-25_	β-lactamase	aminopenicillins	1	1.4
*bla*_SHV-27_	ESBL	aminopenicillins, cephalosporins	1	1.4
*bla*_SHV-36_	β-lactamase	aminopenicillins	1	1.4
*bla*_SHV-37_	β-lactamase	aminopenicillins	1	1.4

### AMR

**Figure 2.**
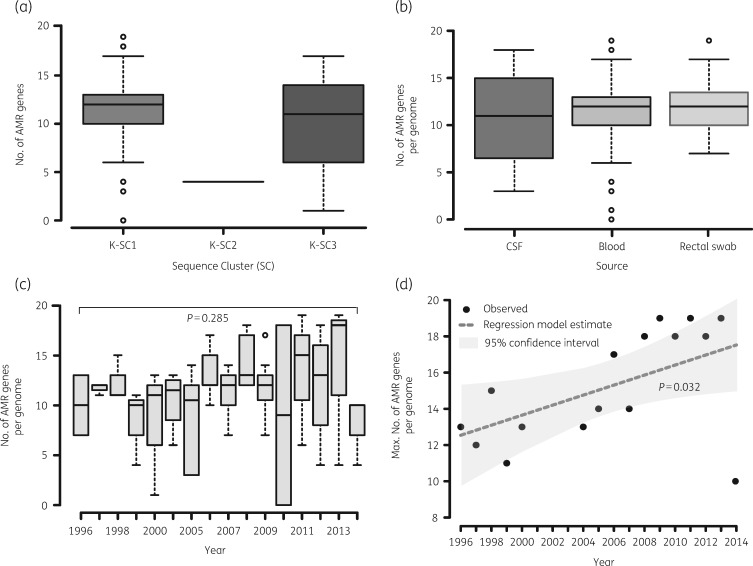
Distribution of number of AMR genes per genome of KPN isolates from Malawi. The figure shows that the median number of genes per genome was similar for KpI (K-SC1) and KpII (K-SC3) isolates but KpIII (K-SC2) genomes carried fewer AMR genes (a). Distribution of genes per genome did not significantly vary based on clinical source of isolation (b) or time (c) but isolates with genomes harbouring higher numbers of genes emerged in the later years (d).

We identified a total of 43 distinct AMR gene alleles, with most genomes having at least 10 AMR gene alleles (Table 3). Distribution of AMR gene alleles by lineage showed that KpI and KpII isolates had relatively similar distributions with both having an average of 11 AMR genes per genome albeit with substantial variations between isolates (range 0–19 for KpI and 0–17 for KpII; Figure 2a). Fewer AMR genes were observed from isolates in KpIII, although this could be due to the low number of isolates in this phylogroup. Both isolates in the KpIII lineage carried four AMR gene alleles, three of which (*fosA*, *oqxA* and *oqxB*) were almost core to the collection (69/72) and a different variant of a *bla*_LEN_ gene for each (Table S1). There were no differences in terms of number of AMR genes per genome based on clinical source (Figure 2c), indicating that carriage isolates are a reservoir of AMR genes. Whilst no significant differences over mean number of AMR gene alleles per genome were observed over time (Figure 2c;*P* = 0.285), there was a significant increase in the maximum number of AMR gene alleles in a genome per year (Figure 2d;*P* = 0.032), which was consistent with the steady increase in phenotypic AMR we have previously reported.^4^

### Molecular determinants of ESBL and FQR

**Figure 3.**
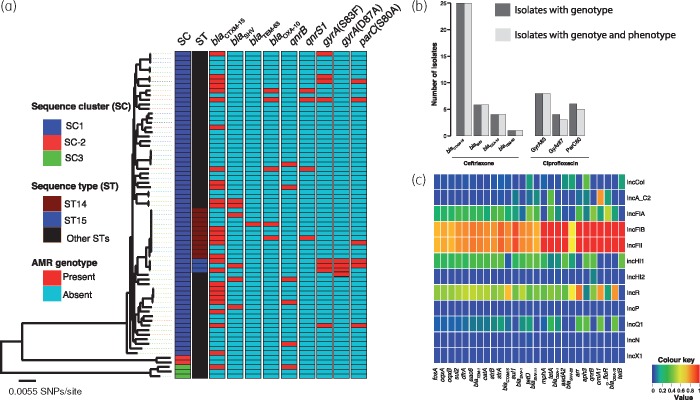
(a) Distribution of ESBL and FQR genotypes across the phylogenetic tree of KPN isolates from Malawi. The figure reveals that except for the *gyrA* mutation at codon position 87, which was associated with ST15, ESBL and FQR genotypes were not restricted to a specific phylogenetic cluster of isolates. (b) Association between isolates with ESBL or FQR genotype and AMR phenotype. (c) A heatmap illustration of associations between plasmid Inc-types and acquired AMR genes present in five or more genomes. Association values were measured as a proportion of the number of isolates with a plasmid Inc-type to the number of isolates with AMR genes. This heatmap shows that most AMR genes of KPN isolates from Malawi were co-occurring with IncFIB and IncFII plasmids. This figure appears in colour in the online version of *JAC* and in black and white in the print version of *JAC*.

Resistance to cephalosporins was conferred by a variety of ESBL-encoding gene variants. Amongst identified ESBL genes, *bla*_CTX-M-15_ was predominant [28/72 (38.9%)] but we also identified *bla*_SHV_, *bla*_OXA-10_ and *bla*_TEM-63_ genes in a number of isolates (Table 3). There was 100% concordance between presence of all ESBL genes and ceftriaxone resistance phenotype (Figure 3b). The *bla*_CTX-M-15_ gene was always associated with plasmid sequences of IncFII and IncFIB types (Figure 3c). The genetic environment of *bla*_CTX-M-15_ consisted of the insertion element IS*Ecp1* upstream of the gene, similar to what we previously observed in *Escherichia coli* from the same setting, raising the possibility of interspecies HGT of this ESBL gene facilitated by the IncFII and IncFIB plasmids.^30^

We screened for amino acid substitutions in the QRDRs of *gyrA*, *parC, gyrB* and *parE* and identified mutations at codon positions S83I (four isolates), S83F (three isolates) and S80Y (one isolate) in the amino acid sequence of *gyrA* and S80I (six isolates) in *parC*, but ciprofloxacin resistance was associated with the *gyrA* mutations (Figure 3b;*P* < 0.001). Except for the *gyrA* mutation D87A, which we only identified in genomes of ST15 isolates, ESBL and FQR genotypes were not strongly linked to a particular lineage of KPN in Malawi (Figure 3a). The amino acid substitution D87A was linked to ST15 isolates, which were all ciprofloxacin resistant.

The collection also contained isolates that had acquired AMR genes associated with low-level FQR including *oqxA/oqxB* [67/72 (93.1%) isolates], *qnrB* [6/72 (8.3%) isolates], *qnrS* [4/72 (5.6%) isolates] and the *aac(6′)-Ib-cr* gene. We found evidence of association between the presence of *qnrB* or *qnrS* genes and ciprofloxacin-resistance phenotype (*P* < 0.0001). In contrast, *oqxA* and *oqxB* were not associated with ciprofloxacin resistance phenotype in this collection (*P* = 0.558). This was not surprising, as the presence of these genes on their own does not necessarily result in resistance, unless overexpressed.^31^

**Figure 4.**
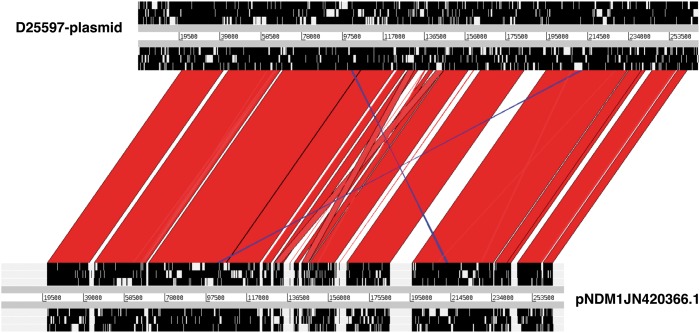
A pairwise comparison of plasmid pNDM1JN420336.1, which harbours the carbapenemase-encoding gene *bla*_NDM-1_, and a plasmid sequence from isolate D25597 from the Malawian KPN collection by the Artemis comparison tool (ACT). Red and blue blocks connect regions that are conserved between the two plasmid sequences in forward and reverse orientations, respectively. Non-conserved regions between the two plasmid sequences are connected by white blocks. The overall level of similarity between the two plasmid sequences was 96.6% at 88.4% coverage. This figure appears in colour in the online version of *JAC* and in black and white in the print version of *JAC.*

The majority of the AMR genes were associated with IncFIB and IncFII plasmids (Figure 3c). The IncFII and IncFIB replicons were identified in exactly the same isolates and were associated with exactly the same genes, suggesting either that these two replicons were on one plasmid or that they each represent different plasmids that coexist to provide stability to each other. No carbapenem resistance genes were detected in the Malawian KPN isolates; however, some of the IncFII and IncFIB plasmid replicons were almost identical (match identity >99.0% and 100.0% coverage) to those of the carbapenem resistance plasmid pNDM-mar (Genbank: JN420336.1). We mapped sequence reads of one isolate (D25597) to the pNDM-mar plasmid and a pairwise comparison of the two plasmid sequences using the Artemis comparison tool (ACT)^32^ showed high similarity (Figure 4).

## Discussion

KPN is a pathogen of global importance due to its association with extensive AMR but little is known about the genomics of this pathogen in sSA.^33^^,^^34^ In order to expand the understanding of the genomics of MDR KPN in sSA, we placed a KPN collection from Malawi in the context of other previously sequenced KPN isolates from Kenya and elsewhere across the globe. We have shown that the KPN population in Malawi fits well into the global population structure of KPN but KpI isolates from sSA exhibited less nucleotide diversity compared with each other than compared with the global isolates. Whilst the reduced nucleotide diversity in the Malawian and Kenyan isolates could reflect the fact that isolates were obtained from single sites, it also suggests that fewer clones are responsible for KPN infections in sSA than is the case globally. In particular, we have identified CC14, consisting of mostly ST14 and a few ST15 isolates, as an important KPN clone associated with invasive disease in Malawi. Within sSA, the predominance of ST14 among invasive KPN isolates is not unique to Malawi, although in the previous studies it was associated with HA infections. ST14 was identified as the most common KPN ST causing HA paediatric infection in Tanzania and was also linked to a hospital outbreak in South Africa.^34^^,^^35^ These findings suggest that KPN ST14 is endemic to sSA. Furthermore, the similarity in nucleotide diversity between the Malawian and Kenyan isolates suggests KPN populations in these two sSA countries are under similar selection pressures.

The spread of MDR and ESBL-encoding genes amongst KPN strains has been associated with the expansion of a limited number of KPN epidemic clones.^15^ In this study, MDR and ESBL production were associated with diverse isolates and there was no single particular AMR profile–lineage combination, although our study was not designed to detect the emergence of epidemic clones. The strong association between the majority of the AMR genes and a limited number of plasmid replicons, mostly IncFII and IncFIB, does suggest that a few plasmids may have a key role in harbouring and disseminating AMR genes. A number of KPN isolates (including ST14) from Malawi had plasmids with high sequence similarity to the pNDM-1 plasmid that harbours the *bla*_NDM-1_-encoding gene. The genetic environment necessary for the acquisition, persistence and dissemination of *bla*_NDM-1_ genes is therefore already present in Malawi, should the evolutionary selection pressure for their emergence, through dysregulated carbapenem use, be brought to bear on this population.

The major limitation of this study is that the isolates from Malawi came from a single site thereby limiting generalizability of the findings of this study to the country or region. However, the similarities of our findings with other studies in sSA^7^^,^^34^ show that our study is more likely representative of the genomic epidemiology of KPN in sSA. In selecting isolates with the aim of enriching for diversity, we lost the ability to estimate prevalence of different STs and to study specific STs in depth. However, this approach has improved the ability to describe the population structure and diversity of KPN associated with MDR in this setting.

We have shown that the KPN population in Blantyre, Malawi faces selective pressures that are similar to other settings in sSA, driving spread of multiple AMR genes, including those for ESBLs, across diverse lineages. The consistency in population structure of Malawian isolates with the global isolates further shows that Malawi is connected to the global exchange of circulating MDR KPN lineages and that these may be causing untreatable infections in a setting with very limited antimicrobial options. The presence of plasmids, which have been associated with carbapenemases globally, is of considerable concern in a context in which carbapenems are starting to be used without a robust culture of antimicrobial stewardship.

## Supplementary Material

Supplementary Data
